# Development and progress for three decades in *umu* test systems

**DOI:** 10.1186/s41021-016-0054-8

**Published:** 2016-12-01

**Authors:** Yoshimitsu Oda

**Affiliations:** Institute of Life and Environmental Sciences, Osaka Shin-Ai College, 6-2-28 Tsurumi, Tsurumi-ku, Osaka 538-0053 Japan

**Keywords:** *umu* test, SOS response, Metabolic activation, Genotoxicity, Cytochrome P450, Glutathione *S*-transferase, *O*-acetyltransferase, Sulfotransferase

## Abstract

*Umu* test have been widely used to predict the detection and assessment of DNA- damaging chemicals in environmental genotoxicity field for three decades. This test system is more useful with respect to simplicity, sensitivity, rapidity, and reproducibility. A review of the literature on the development of the *umu* test is presented in this article. The contents of this article are included a description of numerous data using the *umu* test. This test have been fully evaluated and used in many directions. Different genetically engineered *umu* systems introducing bacterial and rat or human drug metabolizing enzymes into the *umu* tester strains, have been successfully established and are considered as useful tools for genotoxicity assays to study the mechanisms of biotransformation in chemical carcinogenesis. Actually, we developed that two types of bacterial metabolizing enzymes and 4 types of rat and human metabolizing enzyme DNAs are expressed in these strains such as nitroreductase and *O*-acetyltransferase, cytochrome P450, *N*-acetyltransferases, sulfotransferases, and glutathione *S*-transferases, respectively. Due to increasing numbers of minute environmental samples and new pharmaceuticals, a high-throughput *umu* test system using *Salmonella typhimurium* TA1535/pSK1002, NM2009, and NM3009 strains provides a useful for these genotoxicity screening. I also briefly describe the first attempts to incorporate such *umu* tester strain into photo-genotoxicity test.

## Background

Since 1970, a variety of bacterial genotoxicity assays have been developed using *Escherichia coli* and *Salmonella enterica* serovar Typhimurium (*S. typhimurium*) tester strains. They have played an important role in testing and monitoring carcinogenic chemicals, screening novel synthesized compounds, and monitoring environmental samples. In the early 1970s, Ames test, called Salmonella/microsome assay [1, 2] is one of the most useful methods for screening of environmental chemical carcinogens. This test was based on the measure of the reverse mutations from histidine auxotrophy to prototrophy in several constructed Salmonella tester strains.

During 10 years, newly three genotoxicity assays such as the Biochemical prophage induction assay [3], SOS Chromotest [4], and *umu* test [5] have been developed with different principles. The *umu* test is based on the abilities of DNA-damaging agents to induce expression of the *umuC* gene responsible for SOS mutagenesis induced by radiation or chemical agents in *E. coli* [6], the *umuC* gene is regulated by the *lexA* and *recA* genes of bacterial SOS response. In 1982, we began studying on the development of short-term test for detecting environmental mutagens and carcinogens using *S. typhimurium*. We proposed *umu* test in 1985, which is based on a single *S. typhimurium* strain TA1535/pSK1002 harboring a multicopy plasmid pSK1002 with *umuC"* *lacZ* gene fusion [5].

Next, we have developed genetically engineered *umu* tester strains over-expressing bacterial nitoreductase/or *O*-acetyltransfease enzymes for the detection of nitroarenes and arylamines with highly sensitivity [7]. We have further developed genetically engineered *umu* test systems expressing human phase I drug metabolic enzyme (cytochrome P450) [8] and rat or human phase II drug enzymes (glutathione *S*-transferase, *N*-acetyltransfearses, and sulfotransferases) for determination of bioactivation of chemical procarcinogens and promutagens and studies of mechanisms of genotoxicity or carcinogenesis [9–11]. Finally, we recently published our papers on the application of *umu* test to photogenotoxicity [12] and flow cytometry analysis [13].

In this review, I focus on some aspects of the development and progress during three decades regarding our scientific literatures published since 1985 with the genotoxicity assays using *umu* test and their prospects.

### Principle of the *umu* test

When *E. coli* damages DNA or arrests DNA synthesis with ultraviolet light and genotoxins, inhibition of the cell division, prophage induction, DNA repair, and mutagenesis are induced [14]. These cellular functions are called an SOS response [14]. Regulation of the SOS response is mediated through the *recA* and *lexA* genes [15]. The SOS genes consist of approximately 30 unlinked genes [16]. When cells are exposed to chemical carcinogen, an SOS signal is generated and alters RecA protein to an activated form. The activated RecA protein (RecA filament) is facilitated the autocleavage of LexA protein, a repressor of the SOS genes. This autocleavage inactivates the transcriptional repressor activity of LexA, thus leading to induction of the SOS response. After the cell damage is repair, the level of signal drops and RecA protein is no longer activayed. LexA repressor then accumulates and the SOS genes are again repressed under normal condition. This SOS regulation is considered as adaptive response mechanisms to lead a cell survival if repair is completed.

In the event that DNA lesions in *E. coli* cannot be repair accurately, an error-prone replication pathway exists. This pathway, named translesion DNA synthesis (TLS), is the mechanistic basis of SOS mutagenesis [17]. This TLS in *E. coli* depends on the products of the r*ecA* and *umuDC* genes [14]. The *umuDC* genes encode a DNA polymerase (DNA Pol V), able to replicate over abasic sites [18], thymine-thymine cyclobutane dimmers, and pyrimidine-pyrimidone [6–4] photoproducts [19].

**Fig. 1:**
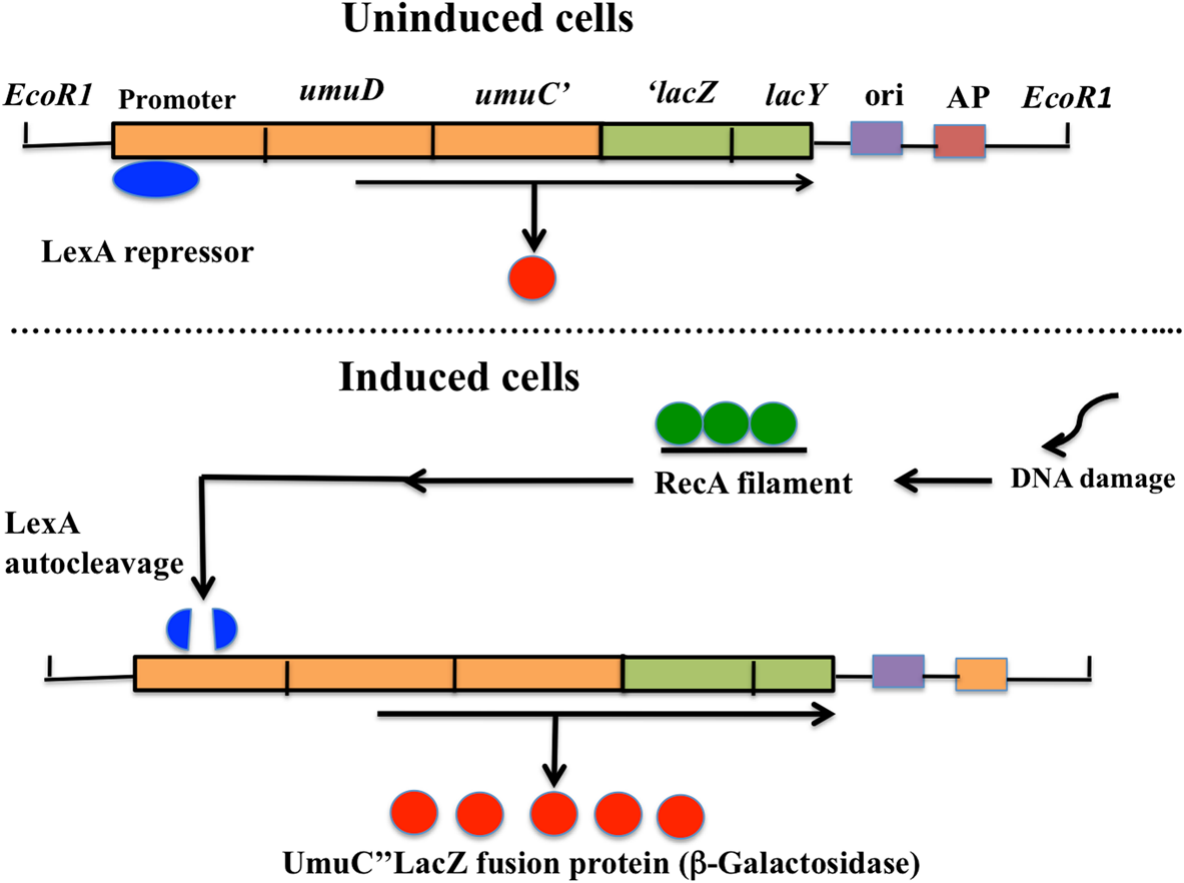
Schema showing the principle of *umu* test using *umuC”lacZ* fusion gene. In uninduced cells, the LexA repressor protein, acts to repress by binding to operator sequences (called an SOS box) upstream from *umu* operon. On the other hand, in induced cells: when the DNA is damaged by genotoxins, or when replication is blocked by various ways, the cell induces an SOS signal. The SOS signal activates a coprotease activity of the RecA protein, and this protease (RecA filaments) activate the autocleavage of the LexA repressor, allowing *umuC*” lacZ fusion gene expression, and the chimeric UmuC”LacZ fused protein is produced. Since the *umuC* gene is fused with the *lacZ* gene for β-galactosidase activity, the induction of *umuC* gene can be estimated by determination of the β-galactosidase activity

The *umuC* gene is controlled by the *recA* and *lexA* genes. Shinagawa et al. [20] constructed by the fusion of *umu* operon to a reporter *lacZ* gene. The *umu* test using *S. typhimurium* TA1535/pSK1002 is assay systems based on a self-cleavage reaction of the LexA representative repressor protein and the fusion of the *umu*-controlled promoter with the *lacZ* gene that can be colorimetrically. The principle of the *umu* test is as followed: when the SOS response is induced by genotoxins, the *umuC*"*lacZ* fused gene which is under the promoter’s control of an *umuDC* gene is expressed, and UmuC"LacZ fused protein of the product is induced. Because this protein has a β-galactosidase activity, it’s possible to check the inductivity of the *umuC* gene expression by measuring this activity. As the result, the DNA-damaging capability due to the chemicals can be supposed easily. Schema of the principle of *umu* test is presented in Fig. 1.

### Development and evaluation of genotoxicity using *umu* test

We first presented the *umu* test in 1982. Our first paper published from Mutation Research in 1985 has been cited about 600 times. In addition, the test strain for *umu* test has been distributed to more than 350 laboratories worldwide so far. We further studied the abilities of 151 chemicals to induce *umuC* gene expression in *S. typhimurium* TA1535/pSK1002 [21]. Data presented that some of the chemicals such as dimethyl sulfoxide, *m*-dioxan, 5-fluorouracil, and paraquat, which have been reported to be non-mutagenic in Ames test, were found out to be positive in *umu* test. Reifferscheid and Heil [22] further compared the results obtained in *umu* test with those obtained in the Ames test for the available data of 486 compounds. The concordance between the *umu* test and Ames test results was about 90 %. In addition, the agreement between carcinogenesis and *umu* response was 65 %. The *umu* test results were highly representative of rodent carcinogenicity [22] (Table 1). Furthermore, Yasunaga et al. [23] examined the genotoxicity of 83 National Toxicology Program (NTP) chemicals including noncarcinogens and carcinogens in the *umu* test. The concordance (67 %) in *umu* test and carcinogenicity test was similar to that (63 %) in the Ames test and carcinogenicity test. Furthermore, the *umu* test has been successfully applied to screen for the presence of genotoxic substances in a broad range of materials and environments such as new drugs, foods, cosmetic products, and working environment as well as to detect the genotoxic effects of radiations and anti-genotoxic compounds so far.

**Table 1:** Comparison of *umu* test results and chemicals tested for rodent carcinogenicity [22]

Carcinogenicity	*umu* test			
	+	—	±	Total
+	119	72	2	193
—	11	39	0	50
±	0	5	0	5
Total	130	116	2	248
Sensitivity	65 %	(119/193)		
Specificity	78 %	(39/50)		
Accuracy	65 %	(158/243)		

In Japan, *umu* test has already been adopted as an official method for water test method in 1993 and the wastewater test method in 1997. Following several modifications, it is used as German standard methods [24] for the examination of water and wastewater testing in 1995. The *umu* test has become the only reporter gene assay to achieve International standardization organization (ISO) standards so far [25]. Similarly, it has been approved as genotoxicity test for wastewater in Malaysian standard method (MS ISO 13829: 2008). An adoptions of the *umu* test for official method was shown in Table 2.

**Table 2:** Standard methods for the determination of the genotoxicity of water and wastewater using *umu* test

Year	Description	Country
1993	An official method of the water supply test method	Japan
1995	A standard method of the genotoxicity of water and wastewater (DIN 38415T3) [24]	Germany
1997	An official method of the wastewater test method	Japan
2000	A genotoxicity test of water and wastewater in International standardization Organization (ISO) Standards (ISO/CD 13829) [25]	ISO
2008	A standard method of the genotoxicity of water and wastewater (MS ISO13829)	Malaysia

Very recently, new electrochemical genotoxicity assays, which enable the analysis of turbid samples, have been developed [26–28]. They are based on the *umu* test using a rotating disk electrode in a microtiter droplet. The results revealed that the signal detection in these assays due to hydrodynamic voltametry was less influenced by the presence of colored components and sediment particles in the samples compared to the usual colorimetric detection.

Brinkmann and Eisentraeger [29] showed that the automated *umu* test is highly applicable for the assessment of non-volatile samples with strong or moderate genotoxic effects using a RoboSeqR 4204 SE pipetting station. In 1986, the *umu* test was first commercialized in the form of a package kit. Very recently, we have developed a new *umu* test kit named as *Umulac AT* ^*R*^ using *S. typhimurium* NM2009 strain (available from Protein Purify Co. Ltd).

### Development of tester strains that can detect nitroarenes and arylamines with high sensitivity

Since bacteria such as *E. coli* and *S. typhimurium* using genotoxicity assays have little capacity for bioactivation of chemicals, the assays are indispensable to the use of exogenous mammalian enzyme systems such as S9 fraction. However, in case of certain classes of nitroarene compounds and arylamines, bacterial enzymes are greatly responsible for the bioactivation. Carcinogenic nitroarenes were activated to genotoxins by reduction to arylhydroxylamine intermediates by bacterial nitroreductase. These arylhydroxylamine derivatives are further activated by *O*-acetyltransferase (*O*-AT) to form the ultimate reactive electrophiles in bacterial or mammalian cell systems [30, 31]. Most of arylamines are metabolized essentially through two steps: *N*-oxydation by cytochrome P450 enzymes, and acetyl coenzyme A-dependent acetylation by *N,O*-acetyltransferase [32, 33]. In 1993, we have improved the sensitivity of carcinogenic nitroarenes and arylamines by making the drug-metabolizing enzyme overproducing in the bacterial cell: I subcloned the nitroreductase (NR) gene or both NR and *O*-AT genes into plasmid vector pACYC184, and developed new tester strains NM2009 and NM3009, which overproduced bacterial *O*-AT and NR/*O*-AT, respectively [7, 34, 35] (Table 3). Among six tester strains, NM3009 showed the highly sensitivity to chemical carcinogens such as 1-nitronaphthalene, 2-nitrofluorene, 3,7-dinitrofluoranthene, 3-nitrofluoranthene, 5-nitroacenaphthene, 2-nitronaphthalene, 1-nitropyrene, 1,6-dinitropyrene, 3,9-dinitrofluoranthene, 4,4′-dinitophenyl, 1,8-dinitropyrene, *m*-dinitrobenzene, 2,4-dinitrotoluene, and 1,3-dinitropyrene. We demonstrated that strain NM3009 enhanced the sensitivity in detecting genotoxic nitroarenes [7] (Table 4). These highly sensitive tester strains provide many advantages for the detection of genotoxic activities of nitroarenes in environmental samples as well as for studies of mechanisms of activation of these compounds.

**Table 3:** Establishment of *umu* tester strains overexpressing bacterial and mammalian metabolic enzymes

*S. typhimurium*	Character	Detection	Reference
NM1011	Nitroreductase-overexpressing	Nitroarenes	[7, 34]
NM2009	*O*-AT-overexpressing	Arylamines	[7, 35, 36]
NM3009	Nitroreductase- and *O*-AT-overexpressing	Nitroarenes, Arylamines	[7, 35, 70, 71]
OY1002/1A1	Human P4501A1 and NPR, and *O*-AT overexpressing	PAH, Arylamines	[8, 70]
OY1002/1A2	Human P4501A2 and NPR, and *O*-AT overexpressing	Arylamines	[8, 51, 70]
OY1002/1B1	Human P4501B1 and NPR, and *O*-AT overexpressing	PAH, Arylamines	[8, 70]
OY1002/2C9	Human P4502C9 and NPR, and *O*-AT overexpressing		[8, 70]
OY1002/2D6	Human P4502D6 and NPR, and *O*-AT expressing		[8, 70]
OY1002/2E1	Human P4502E1 and NPR, and *O*-AT overexpressing	Nitrosoamines	[8, 70]
OY1002/3A4	Human P4503A4 and NPR, and *O*-AT overexpressing	Aflatoxins	[8, 70]
NM6001	Human *N*-acetyltransferase 1 overexpressing	Arylamines, Nitroarenes	[10, 67, 73]
NM6002	Human *N*-acetyltransferase 2 overexpressing	Arylamines, Nitroarenes	[10, 67, 73]
NM7001	Human sulfotransferase 1A1 overexpressing	Arylamines Benzylic alcohols	[11]
NM7002	Human sulfotransferase 1A2 overexpressing	Arylamines	[11]
NM7003	Human sulfotransferase 1A3 overexpressing	Alkenylbenzenes	[11]
NM5004	Rat glutathione *S*-transferase Overexpressing	Dihaloalkenes	[9, 60]

*NPR,* NADPH-P450 reductase; *O-AT, O*-acetyltransferase; *PAH,* polycyclic aromatic hydrocarbon

**Table 4:** Comparison of the sensitivity of NM2009, NM3009, and TA1535/pSK1002 strains to nitroarenes and arylamines [7, 36]

Chemicals	S9	TA1535/pSK1002	NM2009	NM3009
		Minimal concentration (ng/ml)^a^		
1-nitropyrene	−	13	ND	0.2
3-nitrofluoranthene	−	10	ND	0.08
1,3-dinitropyrene	−	0.4	ND	0.1
1,6-dinitropyrene	−	1.4	ND	0.04
3,7-dinitrofluoranthene	−	1.2	ND	0.05
3,9-dinitrofluoranthene	−	1.8	ND	0.06
2-aminoanthracene	+	8,400	400	ND
6-aminochrysene	+	200	260	ND
Glu-P-1	+	100	80	ND
Trp-P-1	+	80	6	ND
MeAαC	+	800	180	ND
MeIQ	+	1	0.009	ND

^a^The concentration of chemicals that induced *umuC* gene expression by twofold over background levels

*ND,* not determined

Since 1995, we demonstrated that the NM2009 having an *O*-AT-overexpressing activity is highly sensitive to carcinogenic arylamines and aminoazo compounds and heterocyclic amines, when compared with the parental strain TA1535/pSK1002 and the *O*-AT-deficient strain NM2000 [36] (Table 4) and revealed that NM2009 strain provides a very useful to detect the genotoxic effects of potential genotoxic arylamines above, which require metabolic activation via the P450/ acetyltransferase systems. Numerous studies have also been reported that *umu* test using liver microsomal P450-linked monooxygenase systems in NM2009 strain allows the analysis of roles of rat and human P450s in the bioactivation of various carcinogens [37–42]. Shimada et al. [43] examined the catalytic properties of human P450 1B1 for carcinogen activation using recombinant P450 1B1 in yeast microsomes. The results indicated that P450 1B1 is involved in the bioactivation of various procarcinogenic chemicals to DNA-damaging products in the *umu* assay using *S. typhimurium* NM2009. They also compared activities of metabolic activation of a number of polycyclic aromatic hydrocarbons (PAHs), and PAH dihydrodiols and other procarcinogens by recombinant human P450 enzymes using *umu* assay. The results supported the importance of P450 1A1 and P450 1B1 in the activation of PAHs and PAH dihydrodiols; other P450 enzymes such as P450 1A2, 2C9, and 3A4 have abilities to catalyze PAH chemicals at much slower rates [44]. Recently, Shimada et al. [45] examined the metabolic activation of PAHs and aryl- and heterocyclic amines to genotoxic products in *S. typhymurium* NM2009 and showed that P450 2A13 and 2A6 were able to activate several of these procarcinogens. The former two enzymes were especially active in catalyzing the activation of 2-aminoanthracene (2-AA) and 2-aminofluorene (2-AF). The results suggested that P450 2A enzymes, as well as P450 family enzymes including P450 1B1, are major enzymes involved in activating PAHs and aryl- and heterocyclic amines as well as tobacco-related nitrosamines.

As deactivation works using *umu* assay, Shimada et al. [46] have studied that the effects of several organoselenium compounds 1,2-, 1,3-, and 1,4-phenylenebis(methylene)selenocyanate (XSCs) as well as inorganic sodium selenite on the activities of xenobiotic oxidation and procarcinogenic activation by human liver microsomes and by recombinant human P450 1A1, 1A2, and 1B1 enzymes using NM2009 strain. The three XSCs were found to be very potent inhibitors of metabolic activation of 3-amino1,4-dimethyl-5*H*-pyrido[4,3-b]indole (Trp-P-1), 2-amino-3,5-dimethylimidazo[4,5-*f*]quinoline (MeIQ) and 2-AA, catalyzed by P450 1A1, 1A2, and 1B1, respectively. These inhibitory effects may, in part, account for the mechanisms responsible for cancer prevention by organoselenium compounds in laboratory animals. In addition, they examined if individual PAHs and other procarcinogens affect the activities of human P450 1A1, and 1A2, 1B1 by measuring 7-ethoxyresorufin *O*-deethylation activity and metabolism activation of PAH dihydrodiols and MeIQ to genotoxic metabolites in *umu* assay. The results revealed that three selected PAHs (5-methylchrysene, B[*a*]P, and B[*a*]A) inhibited metabolic activation of 5-methylchrysene-1,2-diol, (+/−)-B[*a*]P-7,8-diol, dibenzo[*a,l*]pyrene-11,12-diol, and MeIQ to genotoxic metabolites catalyzed by P450 1A1, 1A2, and 1B1 in *S. typhimurium* NM2009 [47]. Recently, we examined the abilities of naturally occurring furanocoumarins such as isoimperatorin, imperatorin, (+)-oxypeucedanin, (+)-byakangelicol, and (+)-byakangelicine to suppress carcinogens- and procarcinogens-induced DNA damages using *umu* assay and also evaluated the abilities of these compounds to inhibit human and rat P450 1A enzymes in vitro [48]. The results suggested that isoimperatorin, imperatorin, (+)-oxypeucedanin, (+)-byakangelicol, and (+)-byakangelicine significantly suppressed 2-[2-(acetylamino)-4-amino-5-methoxyphenyl]-5-amino-7-bromo-4-chloro-2-*H*-bcenzo-triazole- and MeIQ-induced genotoxicities. The mechanism on these anti-genotoxic effects might be due to the inhibition of metabolic activation of procarcinogens catalyzed by P450 1A1 and 1A2. In conclusion, we suggested that SOS activation and deactivation assays using *umu* strains can be evaluated a variety of genotoxic carcinogens in terms of the catalytic specificity of mammalian P450 enzymes toward their activation.

UDP-glucuronosyltransferases (UGTs) are important enzymes that detoxicate many procarcinogens. The procarcinogens, which undergo bioactivation by P450-directed oxidation, become good substrates for the UGTs. To analyze if glucuronidation contributes to the elimination of P450-mediated reactive intermediate metabolites to prevent a toxic event, Yueh et al. [49] examined for their ability of 11 human UGTs to modulate the genotoxic actions of *N*-hydro-2-acetylaminofluorene (*N*-hydroxy-2-AAF) and 2-hydroxyamino-1-methyl-6-phenylimidazo[4,5-*b*]pyridine (*N*-hydroxy-PhIP) formed by P450 1A2 with *umu* assay using *S. typhimurium* NM2009. In the presence of uridine 5′-diphosphoglucuronic acid, UGT 1A9 inhibited the genotoxicity of *N*-hydroxy-2-AAF when incubated at 25 μM and completely abolished the genotoxicity at lower concentrations. However, the genotoxicity of *N*-hydroxy-PhIP did not be interfered by the UGT 1A9. This may be due to the dramatic differences in the formation of UGT 1A9 generated glucuronide.

### Development of a new genotoxicity test system with *umu* tester strains expressing phase I and phase II human drug metabolizing enzymes

Numerous genotoxic compounds are metabolically activated by phase I and phase II drug-metabolizing enzymes (DMEs) to electrophilic species which covalently bind to DNA and produced the genotoxic/mutagenic activity. The DMEs can be classified into two main groups: oxidative or conjugative. The cytochrome P450/ NADPH-cytochrome P450 reductase involved in the phase I drug metabolism first modify these compounds with functional groups by oxidation, reduction and hydrolysis. Furthermore, the phase I intermediates are metabolized by glutathione *S*-transferases, acetyltransferases and sulfotransferases involved in phase II drug metabolism.

### Human cytochrome P450s

In order to develop an alternative method (s) to overcome the species differences and to evaluate bioactivation of chemicals in humans, I first established many new *umu* tester strains expressed phase I human cytochrome P450 monooxygenase (P450). The strain was constructed by introducing plasmid pCW’/1A2: hNPR (carrying cDNAs of P450 1A2 and NADPH-P450 reductase in the isopropyl-*a*-D-thiogalactoside (IPTG)-inducible biocistronic construct) and pOA101 (carrying *umuC”lacZ* fusion gene) into *S. typhimurium* TA1535. The newly developed tester strain *S. typhimurium* OY1001/1A2 was found to activate heterocyclic amines (e.g., 2-amino-3-methylimidazo[4,5-*f*]quinoline (IQ), MeIQ and 2-amino-3,8-dimethylimidazo[4,5-*f*]quiloline (MeIQx)) to reactive metabolites that induce *umuC* gene expression in a concentration-dependent manner without S9 fraction. We demonstrated that the established strain OY1001/1A2 could be of use for the detection of the genotoxicity of arylamines without the addition of metabolic activation enzymes [50]. To further enhance the sensitivity of the strain towards procarcinogenic heterocyclic aromatic amines (HCAs), we developed *S. typhimurium* OY1002/1A2 by introducing pCW”/1A2:hNPR (bicistronic construct co-expressing human P4501A2 and the reductase) and pOA102 (constructed by subcloning the *Salmonella O*-AT gene in the pOA101-expressing *umuC"* *lacZ* gene) in *S. typhimurium* TA1535. In addition, we developed an *O*-AT-deficient strain, the OY1003/1A2, coexpressing human P450 1A2 and reductase. By using strains OY1001/1A2, OY1002/1A2, and OY1003/1A2, we compared the induction of *umuC* gene expression by HCAs and found that the OY1002/1A2 strain was more sensitive than the OY1001/1A2 strain towards HCAs, but not detected with the OY1003/1A2 strain. These results indicated that strain OY1002/1A2 can be used in detecting potential genotoxic arylamines requiring bioactivation by P450 1A2 and *O*-AT [51].

**Fig. 2:**
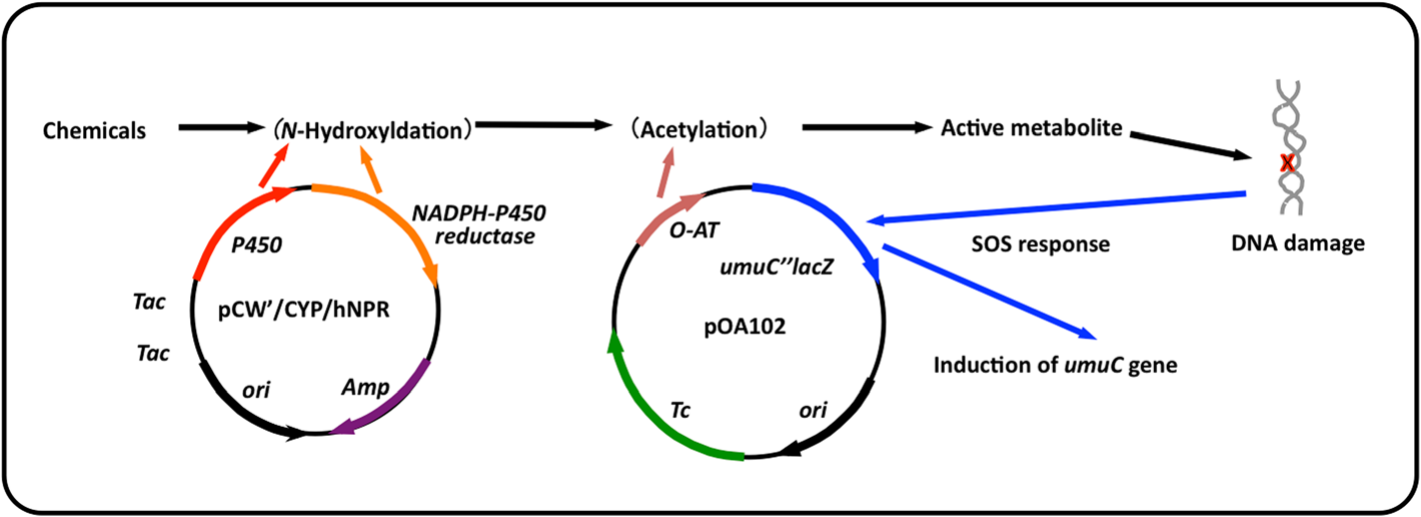
Pathway from metabolic activation to mutagenesis. *P450*, cytochrome P450; *NR,* nitroreductase; *NAT, N*-acetyltransferase; *SULT,* sulfotransferase, *GST* glutathione *S*-transferase

To clarify roles of different P450 enzymes in the bioactivation of HCAs and other procarcinogens, we selected seven of the major human P450 enzymes: P450 1A1, 1A2, 1B1, 2C9, 2D6, 2E1, and 3A4. I further established seven strains OY1002/1A1, OY1002/1A2, OY1002/1B1, OY1002/2C9, OY1002/2D6, OY1002/2E1, and OY1002/3A4 by introducing two plasmids into *S. typhimurium* TA1535, one carrying both P450 and the reductase cDNAs in a bicistronic construct under control of an IPTG-inducible double *tac* promoter and the other, pOA102, carrying *O*-AT and *umuC”lacZ* fusion genes [8] (Table 3). An outline of the *umu* test systems is shown in Fig. 2. Among all homo- and heterocyclic aromatic amines examined, 2-aminoanthracene (2-AA), 2-aminofluorene (2-AF), 2-amino-6-methyl-dipyrido[1,2-*a*:3′, 2′-*d*]imidazol (GluP-1), MeIQx, MeIQ, and IQ showed strong genotoxicity in the OY1002/1A2 strain, and the genotoxicity of IQ and 2-AA was detected in the OY1002/1A1 strain. Aflatoxin B_1_ showed genotoxicity in the OY1002/1A2, OY1002/1A1, and OY1002/3A4 strains. However, β-naphthylamine and B[*a*]P could not detect genotoxicity in any of the strains. These results indicated that the P4501A2 is the major enzyme involved in the metabolic activation of HCAs [8]. These strains could provide a useful tool for studying the roles of human P450 enzymes involved in biotransformation of xenobiotic compounds. Recently, we found that these strains can show the possibility of a high-throughput *umu* test system (under submitted).

Many other researchers have also reported mutagenicity studies using genetically engineered bacterial strains expressing human P450s; Josephy et al. [52] introduced the expression plasmid carrying human P450 1A2 into *S. typhimurium* YG1019 strain to detect the mutagenicity of HCAs and arylamines, and reported that the mutagenicity of 2-AA and 2-AF was detectable with this system. Kranendonk et al. [53] reported on the development of an *E. coli* strain (BMX100), which expressed active human P450 1A2, alone or fused to rat liver NADPH-P450 reductase. Suzuki et al. [54] developed *S. typhimurium* TA1538/ARO strain by introducing an expression plasmid (p1A2OR) carrying human P450 1A2 and the human NADPH-P450 reductase cDNAs and an expression (pOAT) carrying *S. typhimurium O*-AT gene to *S. typhimurium* TA1538 strain to produce the TA1538/ARO strain. TATA1538/ARO strain showed a high sensitivity to mutagenic HCAs with concentration at around picomole order. Also, Kushida et al. [55, 56] developed *Salmonella* tester strains YG7108 2E1/OR and YG7108 2A6/OR highly sensitive to promutagenic *N*-nitrosamines by introducing a plasmid carrying human P450 1A6 and NADPH-P450 reductase cDNAs or human P450 2E1 and OR cDNAs, respectively, into the *ada* ^−^ and *ogt* ^−^ deficient strain YG7108. The YG7108 2E1/OR-expressing strain gives a strong mutagenic response to *N*-nitrosodimethylamine (NDMA), *N*-nitrosodiethylamine (NDEA), *N*-nitrosodipropylamine (NDMA), *N*-nitrosodibutylamine (NDBA), *N*-nitrosopyrolidine (NPYR), and 4-(methylnitrosamino)-1-(3-pyridyl)-1-butanone (NNK), but not *N*-nitrosomethylphenylamine (NMPhA), and *N*-nitrosonornicotine (NNN). On the other hand, the YG7108 2A6/OR-expressing strain could detect *N*-nitrosamines such as NDMA, NDEA, NDPA, NDBA, NMPhA, NPYR, NNN, and NNK. They indicated that human P450 2E1 is mainly involved in the metabolic activation of *N*-nitrosamines with a relatively short alkyl chain(s), whereas P450 2A6 was predominantly responsible for the activation of *N*-alkylnitrosamines possessing a relatively bulky alkyl chain(s). Similarly, Cooper and Porter [57] have constructed two mutagenicity tester strains that co-express full-length human P450 2E1 and P450 reductase in *S. typhimurium*6laking *ogt* and *ada* methyltransferase (YG7104ER, *ogt* ^-^ and YG7108ER, *ogt* ^−^, *ada* ^−^). These strains were very sensitive to nitrosamines with longer alkyl side chains including diethylnitrosamine, dipropylnitrosamine and dibuthylnitrosamine. In conclusion, taking all of these reports, obtained in the last decade into account, the bacterial tester strains expressing human P450s may provide a useful tool to evaluate the roles of P450 on the metabolism of drugs and bioactivation of xenobiotic chemicals in humans.

In addition of P450s as phase I enzyme, following phase II enzymes such as *N*-acetyltransferases, sulfotransferases and glutathione *S*-transferases are known to play important roles in the metabolism of various toxic and carcinogenic chemicals.

### Rat glutathione *S*-transferase

Glutathione *S*-transferases (GSTs) are constitutively expressed in all mammalian tissues. Cytosolic GSTs can be classified into four groups (alpha, pi, mu, and theta) on the basis of structural similarity of isolated genes [58]. Most of the glutathione conjugates are less toxic, but in several cases the enzymes convert dichloromethane and short-chain alkyl halides to unstable and genotoxic glutathione conjugates [58]. I subcloned the fragment of *umu* operon into a multicopy vector plasmid pKK233-2 containing rat GST 5–5 gene. The tester strain *S. typhimurium* NM5004 was developed by introducing the plasmid (pOY100) into *S. typhimurium* TA1535 [9] (Table 3). We compared sensitivity of the NM5004 and the parental strain TA1535/pSK1002 to several dihaloalkenes. The NM5004 strain was found to detect the genotoxicity of ethylene dibromide, 1-bromo-2-chloroethane, 1,2-dichloroethane, and methylene dichloride (CH_2_CI_2_), but TA1535/pSK1002 did not affected [9] (Table 5). This result was very similar to the results reported by Their et al. [59] who the dihaloalkanes are mutagenic in Ames strain TA1535 which expresses rat GST protein. Also, ten chemicals-1,2-dibromoethane, *N*-(2,3-epoxypropyl)phthalimide, 1,3-dichloroacetone, CH_2_I_2_, 1,2-epoxy-3-phenoxypropane, 2,3-epoxypropyl *p*-methoxyphenyl ether, 1-bromo-2-chloroethane, 1-bromo-2,3-dichloropropane, CH_2_BrCl, and CH_2_Br_2_-were found to enhance *umuC* induction in the NM5004 as compared the parental strain [60] (Table 5). Interestingly, we could detect the genotoxicity of CH_2_CI_2_ in the NM5004. However, Simura et al. [61] have reported that CH_2_CI_2_ did not be bioactivated by human GST alpha and pi classes of enzymes. This suggests that theta class GST enzyme might play a pivotal role in the activation of CH_2_CI_2_ rather than other GST enzymes. In contrast, in the case of 1-nitropyrene and 2-nitrofluorene, NM5004 strain showed weaker *umuC* induction than the parental strain. This result indicates that the theta class rat GST 5-5 enzyme also involves in the inactivation of potential environmental carcinogenic chemicals. Recently, CH_2_CI_2_ and 1,2-dichloropropane are widely used as industrial solvents. They are known to cause a novel human bile cancer by a Japanese printing factory to the workers. Therefore, this strain might be able to use for further studies of the role of the GST in human cancer risk such as bile duct.

**Table 5:** Comparison of genotoxicity activities of various chemicals in *S. typhimurium* TA1535/pSK1002 and NM5004 strains ^a^ [9, 60]

Chemicals	NM5004 [GST(+)]	TA1535/pSK1002 [GST(−)]
1,2-dibromoethane	+++	−
*N*-(2,3-epoxypropyl)phthalimide	+++	+
1,3-dichloroacetone	++	+
CH_2_I_2_	++	−
1,2-epoxy-3-phenoxypropane	+	−
2,3-epoxypropyl *p*-methxyphenyl ether	+	−
1-bromo-2-chloroethane	+	−
1-bromo-2,3-dichloropropane	+	−
CH_2_BrCl	+	−
CH_2_Br_2_	+	−
1,2-epoxy-3-(4′-nitrophenoxy)-propane	−	++
2,3-dibromo-1-chloropropane	±	+
1,4-dibromo-2,3-epoxybutane	+	++
1,2-epoxy-3-bromopropane	−	+
1,2-epoxy-3-chloropropane	−	±
1,2,3,4-diepoxybutane	+	+
2,3-dibromopropionaldehyde	+	+
1,4-dibromo-2,3-dihydroxybutane	±	−
1,4-dibromobutane	−	−
1,3-dibromoacetone	+	+
2,3-dibromo-1-propanol	±	±
1,2-epoxy-4-bromobutane	±	±
CH_2_Cl_2_	++	−
1,3-dibromo-2-propanol	−	−
1-bromo-2,3-propanediol	−	−
4-vinylcyclohexene dioxide	−	−
eyclohexene oxide	−	−
1,2-epoxybutane	−	−
1-bromo-2-fluoroethane	−	−

^a^ Potencies of chemicals in *umu* systems were ranked as follows: (−) 0–50; (±) 50–100; (+) 100–250; (++) 250–450; (+++) 450 for *umu* gene expression (units)

### Human *N*-acetyltransferases

Numerous studies have shown that nitroarenes and arylamines are present in environment or occupied places [31]. They are reported to be strong mutagens in bacteria and carcinogens in rodents [31, 62–64]. Human *N*-acetyltransferase (NAT) enzyme NAT1 and NAT2, are known to be polymorphic with rapid, intermediate and slow acetylator phenotypes [65]. To clarify the role of two human NAT1 and NAT2 in the genotoxicity of arylamines and nitroarenes, we established strains NM6001 and NM6002 by introducing human NAT1 and NAT2 cDNAs, respectively, into the parental strain NM6000 (TA1538/1,8-DNP/pSK1002) (Table 3). The human NAT1-expressing strain NM6001 showed higher sensitivity than the human NAT2-expressing strain NM6002 to the cytotoxic and genotoxic effects of 2-nitrofluorene and 2-AF [10]. This result was in good agreement with those reported by Grant et al. [66] who showed that 2-AF exhibited the mutagenic response in a *S. typhimurium* strain expressing human NAT1 in the presence of rat liver S9. In contrast, the NM6002 strain exhibited higher sensitivity than the NM6001 strain to the cytotoxic and genotoxic effects by 1,8-dinitropyrene, 6-aminochrysene and MeIQ. Interestingly, we found that the bladder carcinogenic arylamines 4-aminobiphenyl, 2-acetylaminofluorene, β-naphthylamine, *o*-tolidine, *o*-anisidine, and benzidine are mainly activated by the NAT1 enzyme to produce DNA damage rather than NAT2 [67]. These results suggested that the human NAT strains can be employed for the studies on mechanisms of genotoxicity of a variety of nitroarenes and arylamines, along with the assessment of cancer risk to humans.

In the late 2000s, we have reported the roles of human P450s and human NATs enzymes in the metabolic activation of various carcinogenic chemicals. The β-carboline compounds norharman (9*H*-pyrido[3,4-*b*]indole) and harman (1-methyl-9*H*-pyrido[3,4-*b*]indole) are formed in the pyrolysis of tryptophan and are shown to be present at much higher levels than heterocyclic amines in tobacco condensates and cooked foods [68, 69]. These chemicals showed co-mutagenicity with S9 mixure in the presence of aniline and *o*-toluidine. The resulting aminophenylnorharman (APNH), aminomethylphenylnorharman (AMPNH) and aminophenylharman (APH) found to be produced by coupling of norharman and aniline, norharman and *o*-toluidine, and harman and aniline in the presence of S9 mixture. We examined the genotoxicity of these coupling chemicals using *umu* tester strains established in our laboratory. APNH, AMPNH and APH were found to induce *umuC* gene expression in NAT2-overexpressing strain at much higher rate than the NAT1-overexpressing strain. The genotoxicity of APNH, AMPNH, and APH was also detected in OY1002/1A2 strain, OY1002/1A1 and OY1002/1A2 strains, and in OY1002/1A2 strain, respectively. The results suggested that these chemicals were mainly bioactivated via P450 1A2 and NAT2 [70].

3-Nitrobenzanthrone (3-NBA) is a carcinogenic mutagen existed in diesel exhaust, airborne particulate matter, soil, and water [71]. I first constructed the *S. typhimurium* OY1022 strain by selecting resistant colonies of TA1535NR capable of growing in the presence of 1,8-dinitropyrene to reduce the direct sensitivity to 3-NBA and established *S. typhimurium* strains OY1022/1A1, OY1022/1A2, OY1022/1B1, and OY1022/3A4 expressing four recombinant human P450s by introducing two plasmids into the OY1022, one carrying both P450 and NPR cDNAs in a biocistronic construct under control of an IPTG-inducible double *tac* promoter and the other, pOA102, carrying *O*-AT and *umuC"* *lacZ* fusion gene. Using these strains, we investigated whether any human P450 enzymes are involved in the bioactivation of 3-NBA to genotoxic metabolites. 3-NBA was found to induce *umuC* gene expression in OY1022/1A1, and OY1022/3A4 strains and, to lesser extent, OY1022/1A2 and OY1022/1B1 strains, at a much higher rate than the parental OY1022/pCW strain. We demonstrated that the activation of 3-NBA can be catalyzed by human P450 3A4, 1A1, 1A2, and 1B1 and NPR to a genotoxin in the presence of bacterial *O*-AT, probably due to nitroreduction [72].

2-Phenyl benzotriazole (PBTA)-type compounds (such as PBTA-4, PBTA-6, PBTA-7, and PBTA-8) were identified as major mutagens in blue cotton/rayon-absorbed substances collected at sites below textile dyeing factories or municipal water treatment plants treating domestic water and effluents from textile dyeing factories in several rivers in Japan [73]. We examined the genotoxicity of four PBTA derivatives using parental strain TA1535/pSK1002 and *O*-AT-overexpressing strain NM2009. Four PBTA derivatives induced the *umuC* gene expression more strongly in the bacterial *O*-AT-overexpression strain than the parental strain. We also determined the bioactivation of these chemicals by recombinant human or rat P450 enzymes in NM2009. The results showed that human recombinant P450 1A1 enzyme was much more active than P450 1A2 and 3A4 in the genotoxic activation of all PBTA compounds. We further investigated the potential role of human NATs in the activation of them using NM6000, NM6001, and NM6002. PBTA-4 showed almost similar sensitivity in the NAT1-expressing strain and the NAT2-expressing strain, although NAT2-expressing exhibited relatively higher sensitivity to PBTA-6, PBTA-7, and PBTA-8 than NAT1-expressing strain. These results suggested that P450 1A1 and NATs are important enzymes responsible for bioactivation of PBTA-type compounds [74].

3,6-Dinitrobenzo[*e*]pyrene (DNBeP) is a potent mutagen identified in surface soil in two metropolitan area of Japan [75]. Using a variety of *umu* tester strains expressing human P450s and NAT enzymes, we examined the role of human P450 enzymes in the bioactivation of DNBeP to genotoxic metabolites. The dose-dependent induction of *umuC* by DNBeP was observed at concentrations between 0.01 and 1 nM in the *O*-AT-expressing strai, but not in the *O*-AT-deficient strain. In the P450 3A4-, P450 1A2-, P450 1A1-and P450 1B1-expressing strains, DNBeP was found to be activated to reactive metabolites that cause the induction of *umuC* gene expression compared with the parental strain. The induction of DNBeP in the NAT2-expressing strain had a 10-fold lower concentration than that in the NAT1-expressing strain. We suggested that nitroreduction by human P450 1A2, P450 3A4, and P450 1A1 and *O*-acetylation by human NAT2 contributes to the bioactivation of DNBeP [76].

### Human sulfotransferases

Sulfonate conjugation has been shown to be an important pathway in the biotransformation of numerous xenobiotics and endobiotics such as drugs, chemical carcinogens, hormones, bile acids, neurotransmitters, peptides, and lipids [77]. Sulfotransferases (SULTs) transfer the sulfate moieties from the cofactor 3′-phosphoadenosine-5′-phosphosulphate (PAPS) to nucleophilic groups of their substrates. In the case of most xenobiotics and small endogenous substrates, sulfonation has generally been considered as a detoxification process leading to more water-soluble products and thereby facilitating their excreation via kidney or bile [78]. However, for xenobiotics such as *N*-hydroxy arylamines, *N*-hydroxy heterocyclic amines, hydroxymethyl polycyclic aromatic hydrocarbons, the enzymes activate them to highly reactive sulfate esters that bind covalently to DNA [79]. In humans, SULTs consist of four familes, namely SULT1, 2, 4 and 6 and contain at least 13 members of proteins [80]. SULT 1A1, 1A2, 1A3, 1C2, 1E1, and 2A1 are the major enzymes to catalyze the conjugation of xenobiotic chemicals including carcinogens [81].

We developed a newly *umu* assay system to investigate the roles of three different human SULTs, namely SULT 1A1, 1A2, and 1A3, in the bioactivation of aromatic amines, nitroarene compounds, benzylic and allylic alcohols, and estrogens-like compounds to genotoxins [11]. In order to express the three different SULT enzymes in *S. typhimurium,* I subcloned human SULT 1A1, 1A2, and 1A3 cDNA genes into the multicopy plasmid vector pTrc99A^KM^. The generated plasmids were introduced into the *S. typhimurium O*-AT-deficient strain NM6000 (TA1538/1,8-DNP/pSK1002), resulting in the tester strains NM7001, NM7002, and NM7003 (Table 3). These test systems are highly sensitive for SULT-dependent carcinogens without external supply of the cofactor PAPS and MgSO_4_. We compared the sensitivities of three strains with the parental strain NM7000 against 51 chemicals with and without S9 mix. 2-Amino-3-methyl-9*H*-pyrido[2,3-*b*]indole (MeAαC) and Glu-P-1 exhibited strong genotoxicity in the strain NM7001 in the presence of liver S9 mix compared with the strains NM7002, NM7003 and NM7000 (Table 5). The results were consistent with Glatt et al. [82] who reported that MeAαC showed strongly enhanced mutagenicity in a *S. typhimurium* strain expressing SULT 1A1 in the presence of rat liver postmitochondrial fraction compared with a control strain. Furthermore, in the case of Glu-P-1, Chu et al. [83] showed that *N*-hydroxy-Glu-P-1 was selectively sulfonated by a human liver thermostable phenol SULT purified from human liver, probably SULT 1A1 or a mixture of SULT 1A1 and 1A2. These results suggested that human SULT 1A1 is involved in the bioactivation of MeAαC and Glu-P-1 to genotoxic metabolites. On the contrary, 2-AA, 2-acetylaminofluorene, and 2-amino-1-methyl-6-phenylimidazo[4,5-*b*]pyridine (PhIP) exhibited stronger genotoxicity in the strain NM7002 compared with the strains NM7001 and NM7003. The results were in agreement with reports by Glatt and colleague, suggesting that the *N*-hydroxy-2-acetylaminofluorene is activated most efficiently by SULT 1A2 expressed in *S. typhimurium* [84]. Arylamines such as 2-AA, 4-aminobiphenyl, APNH, and 3-methoxy-4-aminoazobenzene showed a similar genotoxic potential in strains NM7001 and NM7002, suggesting that these chemicals are bioactivated by SULT 1A1 and 1A2. NM7001, NM7002, and NM7003 strains were found to be of similar sensitivities toward 2-amino-9*H*-pyrido[2,3-*b*]indole and β-naphthylamine. In cases of 6-aminochrysene, MeIQ, Trp-P-1, and 3-amino-1-methyl-5*H*-pyrido[4,3-*b*]indole, all strains used showed similar sensitivities (Table 6).

**Table 6:** Comparison of substrate specificity of human sulfotransferases expressed in *S. typhimurium* TA1538/1,8-DNP/pSK1002 towards a variety of chemicals [11]

Chemicals	S9	SULT isoforms
Arylamines		
2-Aminoanthracene	+	1A1 = 1A2
2-Aminofluorene	+	1A2
2-Acetylaminofluorene	+	1A1 < 1A2
4-Aminobiphenyl	+	1A1 = 1A2
6-Aminochrysene	+	SR
Aminophenylnorharman	+	1A1 < 1A2
AαC	+	1A3 = 1A2 < 1A1
Glu-P-1	+	1A1
IQ	+	SR
MeAαC	+	1A2 < 1A1
MeIQ	+	SR
3-MeO-AAB	+	1A1 = 1A2
β-Naphthylamine	+	1A3 < 1A2 = 1A1
PhIP	+	1A1 < 1A2
Trp-P-1	+	SR
Trp-P-2	+	SR
Nitroarenes		
Furylfuramide	−	1A2
5-Nitroacenaphthene	−	1A1
3-Nitrobenzanthrone	−	1A2 < 1A1
2-Nitrofluorene	−	1A2
Nitrofurazone	−	SR
3-Nitrofluoranthene	−	1A1
1-Nitronaphthalene	−	SR
1-Nitropyrene	−	1A2
2-Nitropropane	−	1A2
4-Nitroquinoline 1-oxide	−	SR
2-Nitrotriphenylene	−	SR
4,4′-Dinitrobiphenyl	−	1A1 = 1A2
3,7-Dinitrofluoranthene	−	1A2 < 1A1
3,9-Dinitrofluoranthene	−	1A2 < 1A1
1,6-Dinitropyrene	−	SR
Benzylic and allylic alcohols		
Estragole	+	1A3
Hycanthone	−	1A1 = 1A3
1′-Hydoxysafrole	−	1A3
1-Hydroxymethylpyrene	−	1A3 < 1A2 < 1A1

SR presents same response in all strains

*AαC*, 2-amino-9*H*-pyrido[2,3-*b*]indole; *Glu-P-1,* 2-amino-6-methyl-dipyrido[1,2-α:3′,2′-*d*]imidazole; *IQ,* 2-amino-3-methylimidazo[4,5-*f*]quinoline; *MeAαC,* 2-amino-3-methyl9H-pyrido[2,3-*b*]indole; *MeIQ,* 2-amino-3,5-dimethylimidazo[4,5-*f*]quinoline; *3-MeO-AAB,* 3-methoxy-4-aminoazobenzene; *PhIP,* 2-amino-1-methyl-6-phenylimidazo[4,5-*b*]pyridine; *Trp-P-1,* 3-amino-1,4-dimethyl-5*H*-pyrido[4,3-*b*]indole; *Trp-P-2,* 3-amino-1-methyl-5*H*-pyrido[4,3-*b*]indole

Of the 15 nitroarenes, 5-nitroacenaphthene, 3-nitrobenzanthrone (3-NBA), and 3,9-dinitrofluoranthene showed the highest genotoxic potential in the strain NM7001 (Table 6). Arlt et al. [85] reported that human SULT 1A1 is involved in the formation of DNA adducts by 3-NBA using Chinese hamster lung cell line that expresses human SULT 1A1. This finding is consistent with our results that 3-NBA is bioactivated by human SULT 1A1.

The strain NM7002 was highly sensitive to 2-nitrofluorene, 1-nitropyrene and 2-nitropropane. However, in the case of other nitroarenes such as furylfuramide, 3-nitrofluoranthene, nitrofurazone, 1-nitronaphthalene, 4-nitroquinoline 1-oxide, 2-nitrotriphenylene, 3,7-dinitrofluoranthene, and 1,6-dinitropyrene, the genotoxicity was almost equal in all strains (Table 6).

Among numerous benzylic alcohols, 1′-hydroxysafrole and estragole were strongly activated in the strain NM7003 that expresses the human SULT 1A3 (Table 6). The result was the first evidence that human SULT 1A3 plays an important role in the metabolic activation of benzylic alcohols to genotoxic intermediates.

Finally, we showed as well that the genotoxic potency of several chemicals is reduced by SULT enzymes. For example, the genotoxicity of Glu-P-1, PhIP, 2-nitrofluorene, 3-nitrofluoranthene, 1-nitropyrene, and 3,7-dinitrofluoranthene was inhibited by SULT 1A3. In the case of acrolein, the genotoxicity was inhibited by SULT 1A1 and 1A3. These findings suggested that SULT 1A1 or SULT 1A3 enzymes were involved in the detoxification of several genotoxic compounds. The *umu* test system with over-expressed human SULT enzymes may provide to be useful for a further investgation of the SULT-function in the metabolic inactivation of carcinogens.

In summary, using these strains exhibiting phase II human NATs as well as SULTs, we demonstrated that these assay systems provides a sensitive means of assessing the genotoxicity of procarcinogens requiring activation by these enzymes, and useful tools for studying the role of human drug enzymes in biotransformation of xenobiotic chemicals.

### Development of a high-throughput *umu*-microplate test system

Because chemical mutagens and carcinogens are present in the environment in minute quantities, the development of small-scale, rapid and sensitive bioassay system is required for the detection of these environmental genotoxines. We newly developed a rapid *umu*-microplate test system that used *S. typhimurium* strains TA1535/pSK1002, NM2009, and NM3009 to detect genotoxic activity in small-volume samples. The results indicated that the genotoxicity was detected mainly in the fine fraction but also partially in the coarse fraction. The pattern of the response suggested that the genotoxic activity of the particulate extract was due primarily to nitrated polycyclic aromatic hydrocarbons. As an application of the assay, we demonstrated that the assay could be determined the genotoxicity of atomospheric paticulate extracts and the microplate test assay may be useful tool for genotoxicity in small-volume environmental samples [86, 87]. As other examples, Ma et al. [88] performed in conjunction with analytical measurements to identify potential genotoxins in river and adjacent ground waters in Jialu river basin, China. The genotoxicity was identified by using LC-MS/MS analysis that flumequine was one of the causal agents. In addition, the specific response to NM3009 compared with TA1535/pSK1002 demonstrated the presence of nitroarenes in the river sample, although the extract chemicals could not be identified by analyzing the potential nitroarenes commonly detected in the environment. Since the identification of major putative genotoxic compounds in most surface waters with high genotoxic activity in the world has not been performed, further efforts on chemical isolation and identification by bioassay-directed chemical analysis should be performed. Recently, Tian et al. [89] evaluated the applicability of BugBuster Master Mix (B. M. mix) for *umu* test to compare the performance with that of the sodium dodecyl sulfate-Z-buffer system in detecting the genotoxicity of some pure chemicals and various environmental water samples. The results indicated that B. M. Mix, as an effective enzyme extraction reagent, could increase the sensitivity of the *umu* test. Ozonation was also found to be effective in removing genotoxicity from wastewater, whereas chlonination of the reclaimed water led to the increase of genotoxicity.

### Application of *umu* test to photogenotoxicity and flow cytometry analysis

An increasing number of compounds are proven to be photo-genotoxic, although the mechanism of action on DNA varies with the compounds. The photo-genotoxicity can now be detected by various assay systems [90]. I established tester strains NM8001 and NM8021 by introducing the pSK1002 plasmid into strains YG3001 and YG3002, respectively. We selected six compounds that are known to be photo-genotoxic [8-methoxypsoralen (8-MOP), chlorpromazine (CPZ), methylene blue (MB), neutral red (NR), dichlorobenzidine (DCB), 9,10-dimethylbenzanthracene (DMBA)] and evaluated them using the 96-well protocol of the *umu* test after UVA irradiation with the original strain *S. typhimurium* TA1535/pSK1002 as well as NM8001 and NM8021 [12]. The latter two strains are highly sensitive to oxidative DNA damage owing to the deletion of the nucleotide excision repair enzyme *uvrB* and the base excision repair enzyme *mutY*, and the nucleotide excision repair enzyme *uvrB* and the base excision repair enzymes *mutY* and *mutM*, respectively. Among the compounds tested under UVA irradiation, 8-MOP, CPZ, and DMBA showed a significant induction of β-galactosidase activity in NM8001 and NM8021 strains whereas MB, NR, and DCB showed only a slight increase in the β-galactosidase level. The activity of NM8001 induced by the photo-genotoxins was quite similar to that of NM8021, suggesting that the deficiency of *mutY* did not affect detection of the selected photo-genotoxins. These results indicated that the photo-irradiated 96-well version of the *umu* test can be used for rapid screening of the photo-genotoxicity of compounds.

Atmospheric and room temperature plasma (ARTP) mutagenesis has been used for the mutation breeding of more than 40 microorganisms. However, ARTP mutagenesis has not been quantitatively compared with conventional mutation methods. Recently, we developed *umu* test using a flow-cytometric analysis to quantify the DNA damage caused by ARTP, UV, and chemical (4-NQO and MNNG) treatments in individual viable cells using *S. typhimurium* NM2009 as the model strain and to determine the mutation rate [13]. The mutation rate was found to be proportional to the corresponding SOS response induced by DNA damage. Although the conventional *umu* test was used to detect the genotoxicity to both dead and viable cells, regardless of the type of DNA damage, in this study, we concluded that *umu* test using flow cytometry can quantitatively measure DNA damage with viable cells.

## Conclusion

In this review, I described that the *umu* test has been used for detecting potential DNA-damaging agents during three decades. The main advantages of this test may be practical. A single strain *S. typhimurium* TA1535/pSK1002 is used. A quantitative colorimetric response is completed in a few hours. The manipulations are performed with very simplicity since only test tubes or microplates are required and need not be done under strict sterile conditions. In addition, the *umu* test can use for the detection of strong cytotoxic compounds to bacteria such as antineoplastic drugs (bleomycin, doxorubicin, mitomycin C). Furthermore, this test is applicable to the kinetic analysis of enzymatic activation of chemical carcinogens by several drug-metabolizing enzymes. Moreover, it may be a useful complement to the Ames test for screening of genotoxic carcinogens. We have established numerous *umu* tester strains by introducing various genes encoding metabolic enzymes for genotoxic bioactivation, including bacterial NR and *O*-AT, phase I enzymes human P450s, phase II enzymes rat GST, human NATs and SULTs. However, although the modification of *umu* test systems expressing mammalian metabolic enzymes has been mentioned in above sections, there still is much room for further improvements in the *umu* test system. Several genes have been ever introduced into the *umu* assay systems. For example, co-expression systems in the same cell for P450s and other enzymes involved in the metabolic activation and inactivation of genotoxins might be developed for the estimation of the mechanistic roles of the enzymes. They are also appropriate for a high-throughput genotoxicity screening. For example, hydroxylation of arylamines and heterocyclic amines and their subsequent *O*-acetylation or sulfation may be carried out by catalysis of P450 1A2 and NAT1/2 or SULT 1A1/1A2 enzymes. These test systems could provide valuable screening tools for genotoxicity in numerous fields and also be appropriate for basic studies on the possible roles of respective enzymes. In addition, these systems have the following many advantages: they are simple, rapid, small sample volumes with a few μl, and are ideally suited for high-throughput applications. Thus, *umu* test systems could be able to use for validation of genotoxicity assays as well as for studying of the mechanisms of biotransformation of chemicals that may be genotoxic to humans.
